# Supplementary figures and images for: Rapid Oxford Nanopore Technologies MinION Sequencing Workflow for Campylobacter jejuni Identification in Broilers on Site—A Proof-of-Concept Study
Source: Animals (Basel). 2022 Aug 13;12(16):2065. doi: 10.3390/ani12162065 (PMC9405271; doi:10.3390/ani12162065)

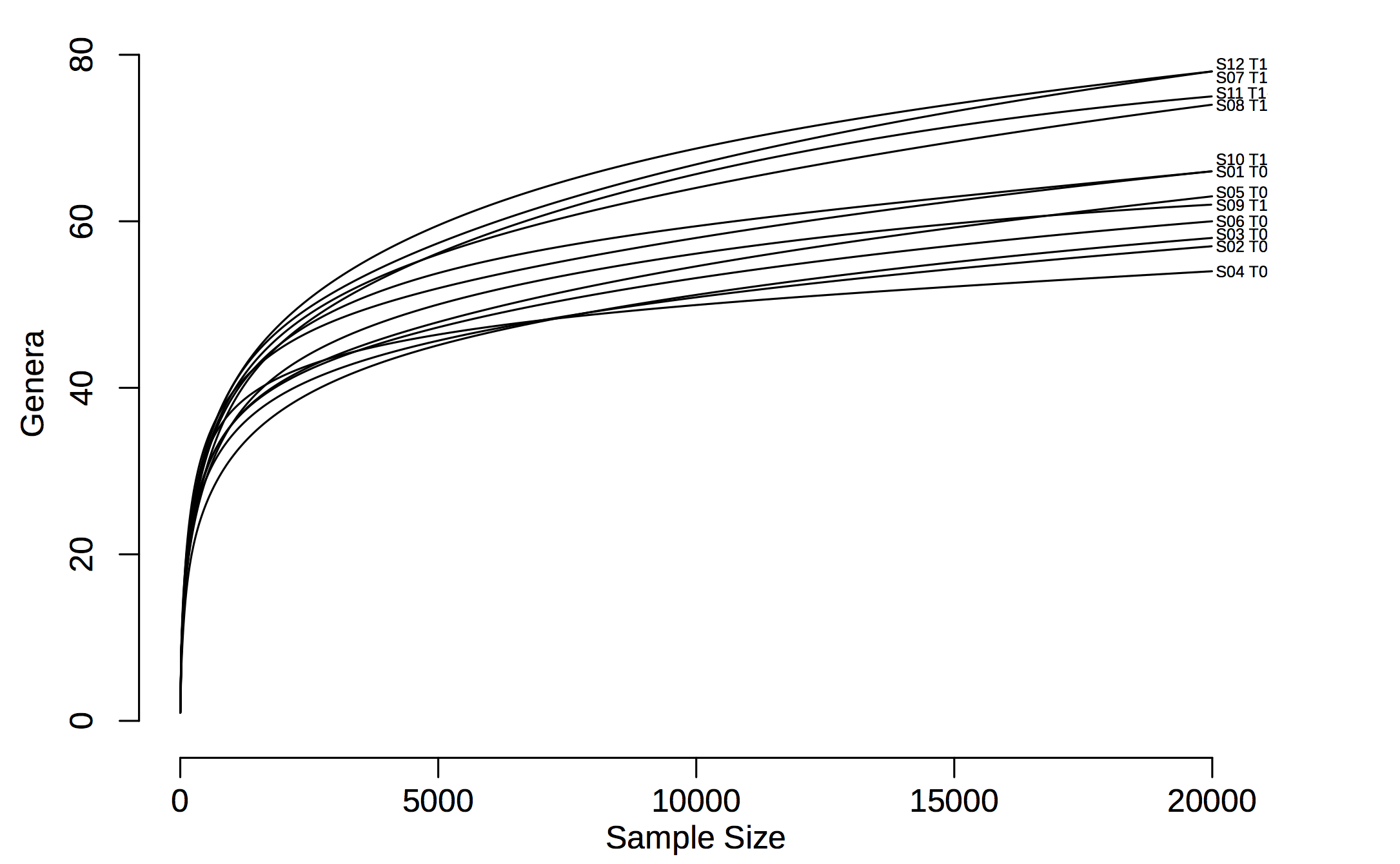

Supplement: Supplementary file 1 [file animals-12-02065-s001.zip › animals-1748653-supplementary.tiff]
